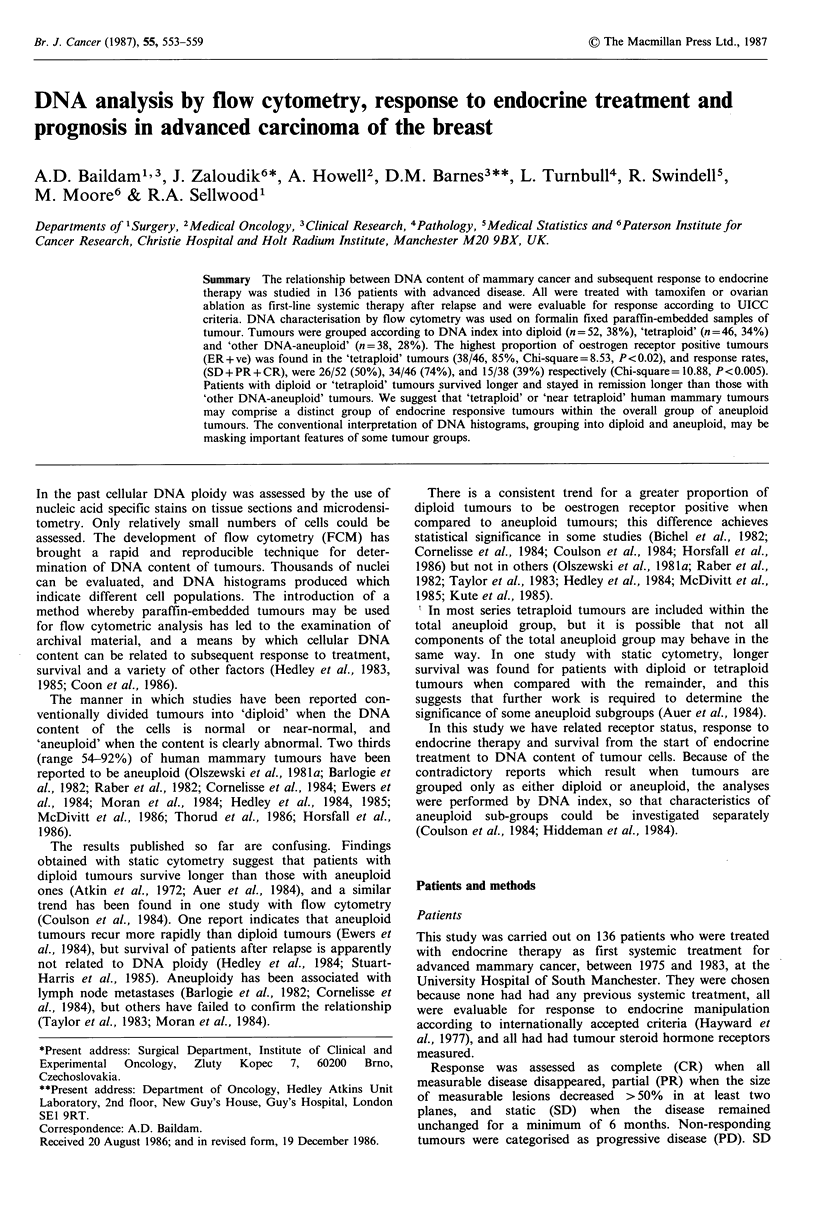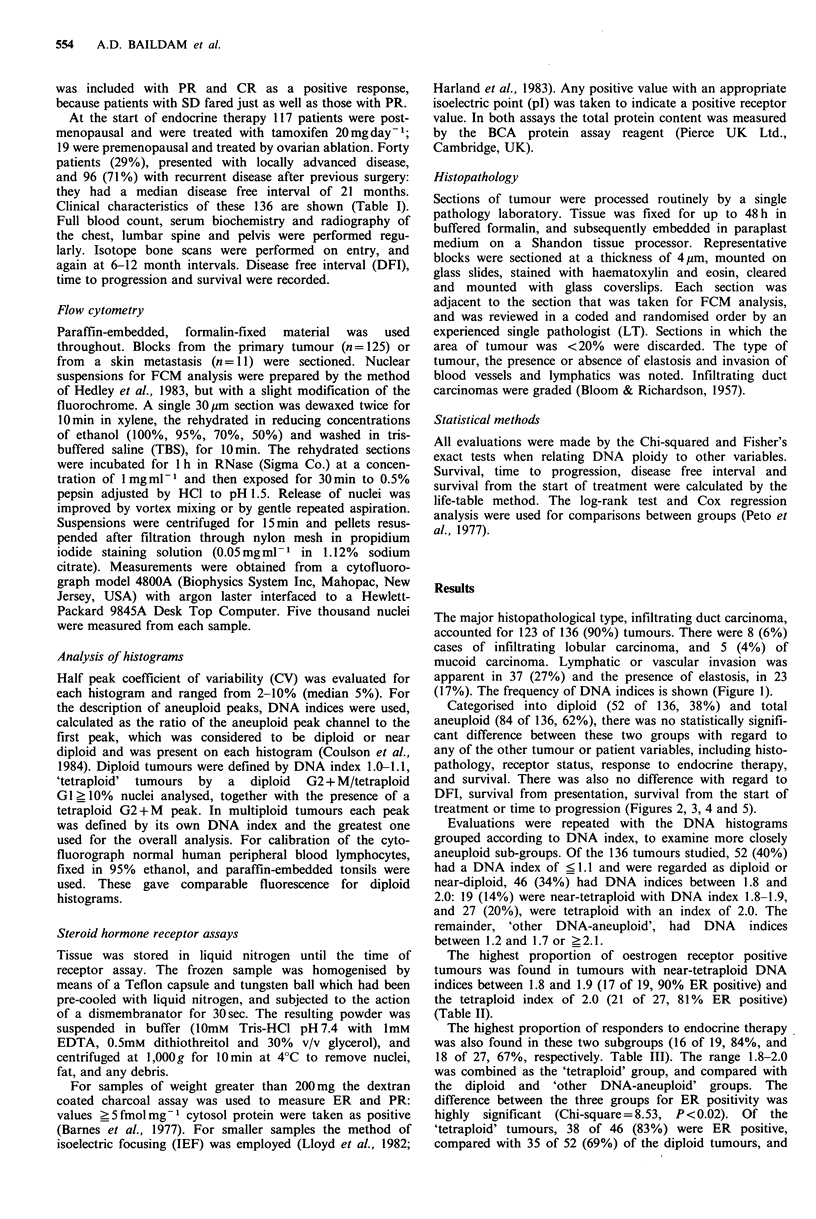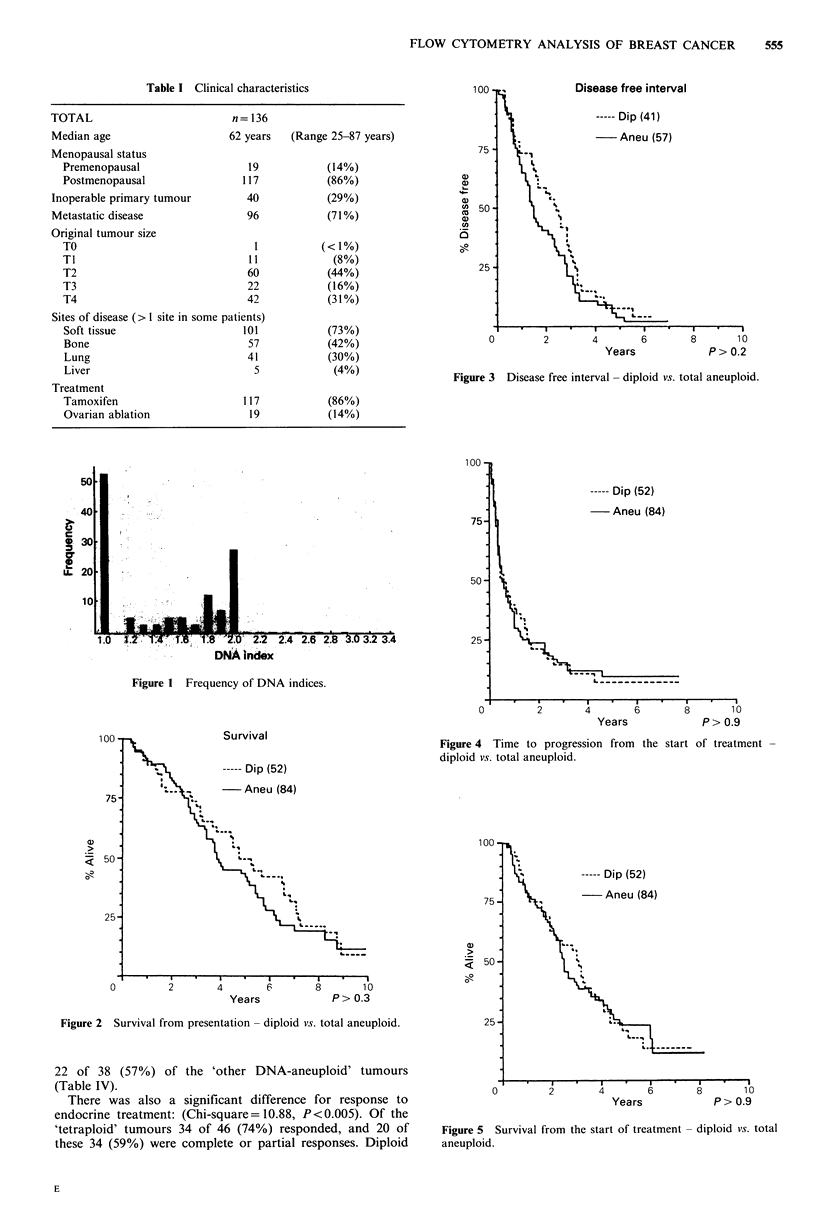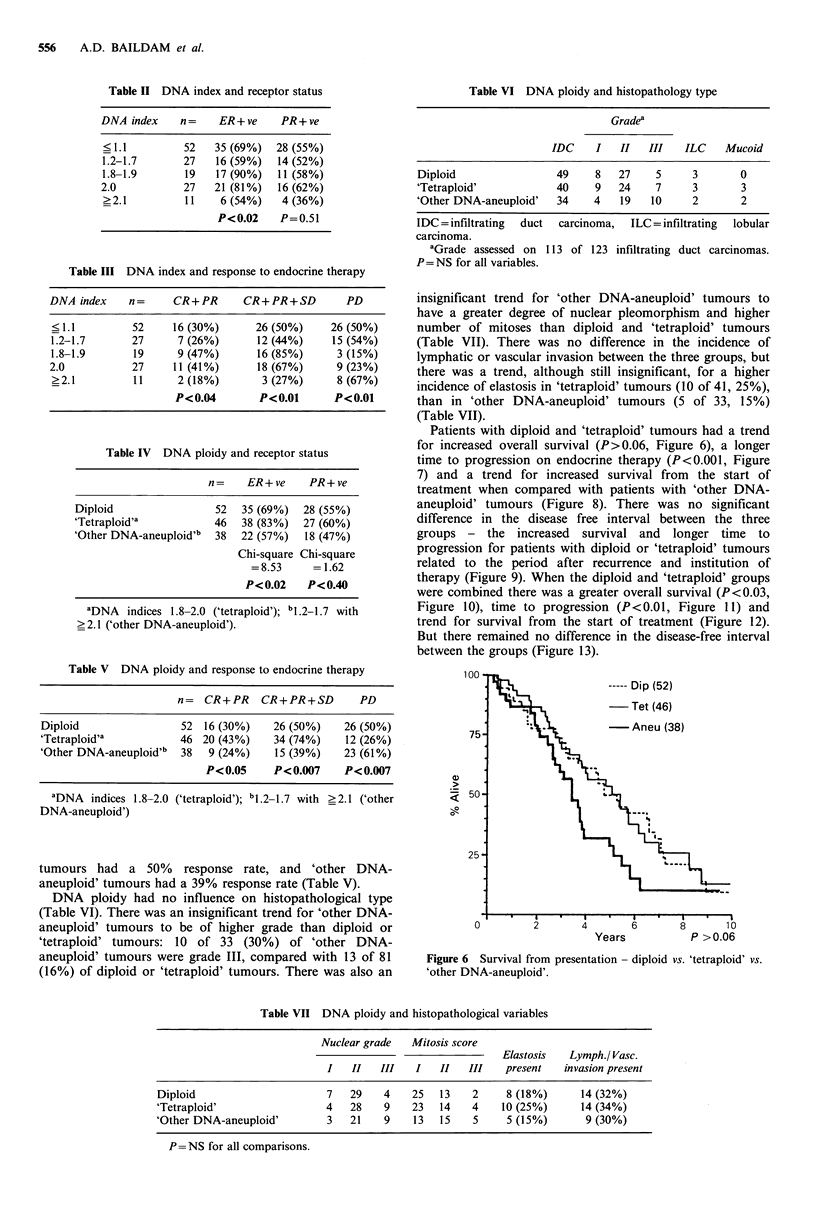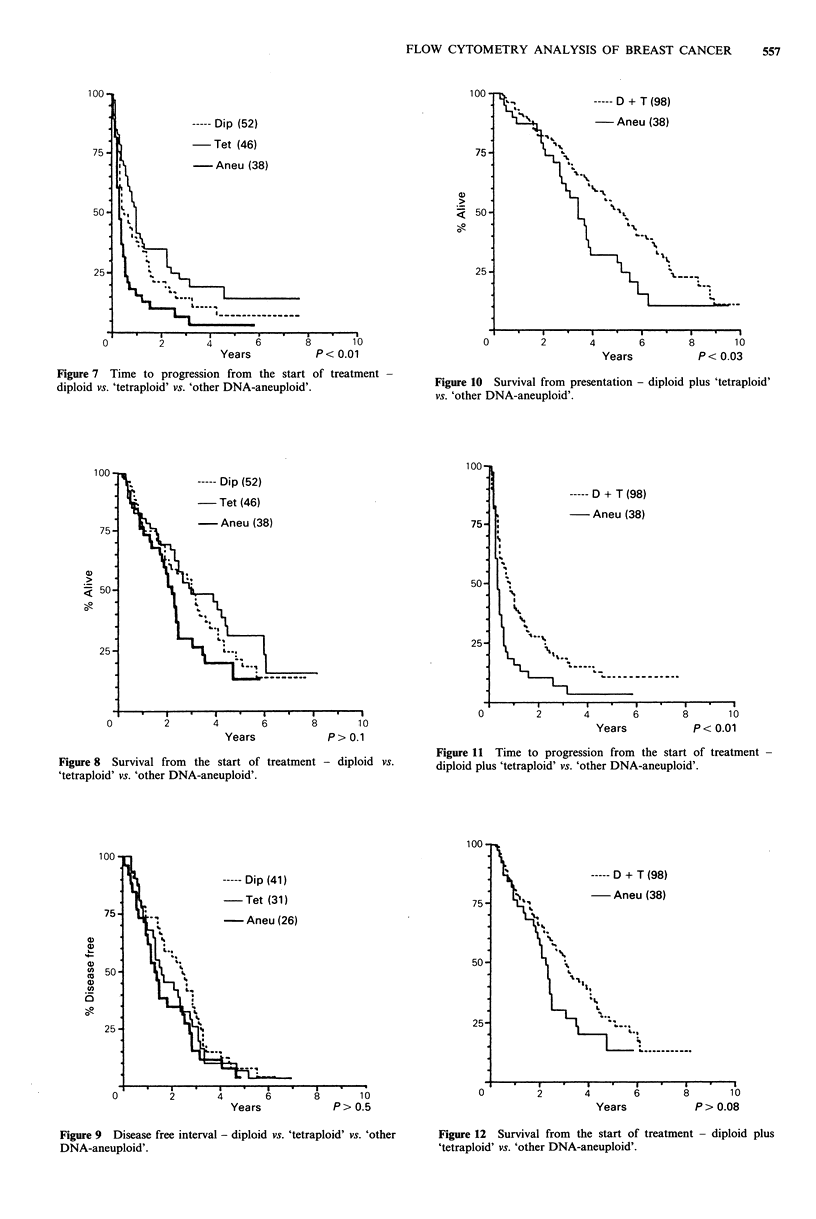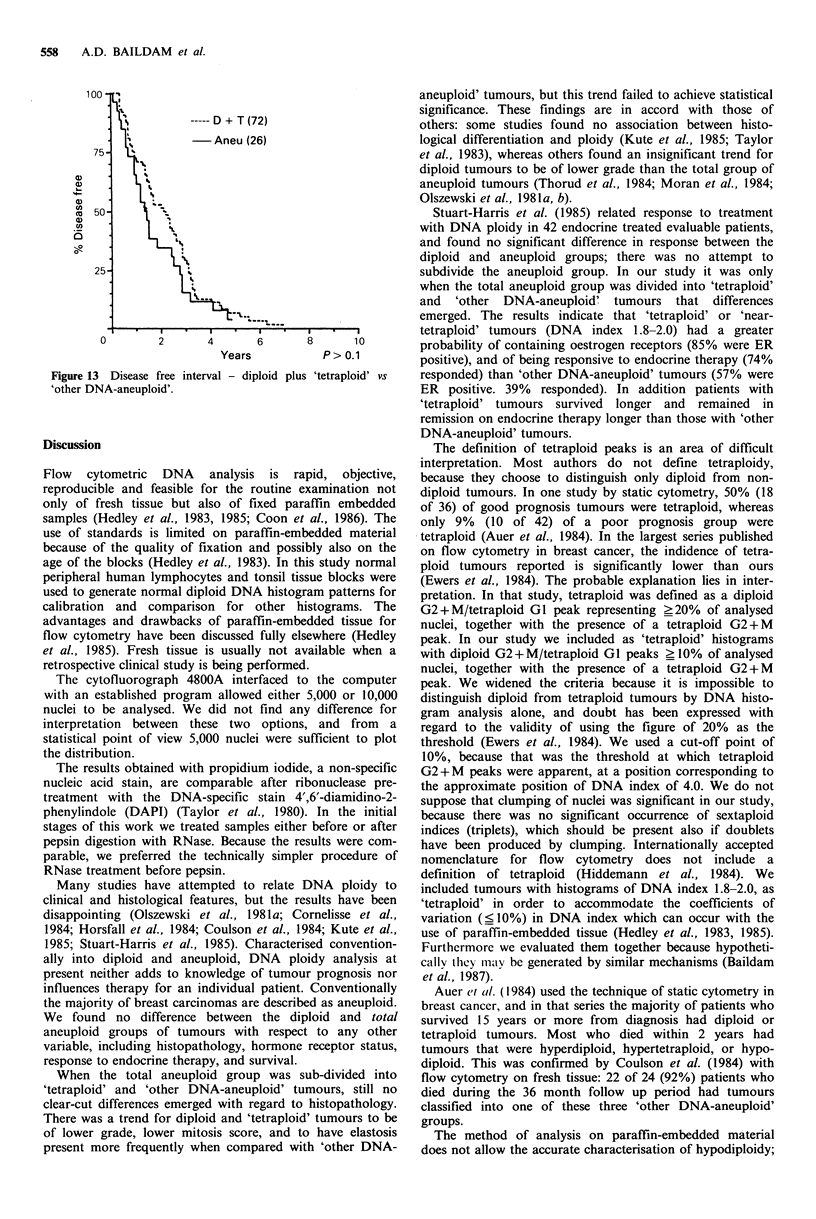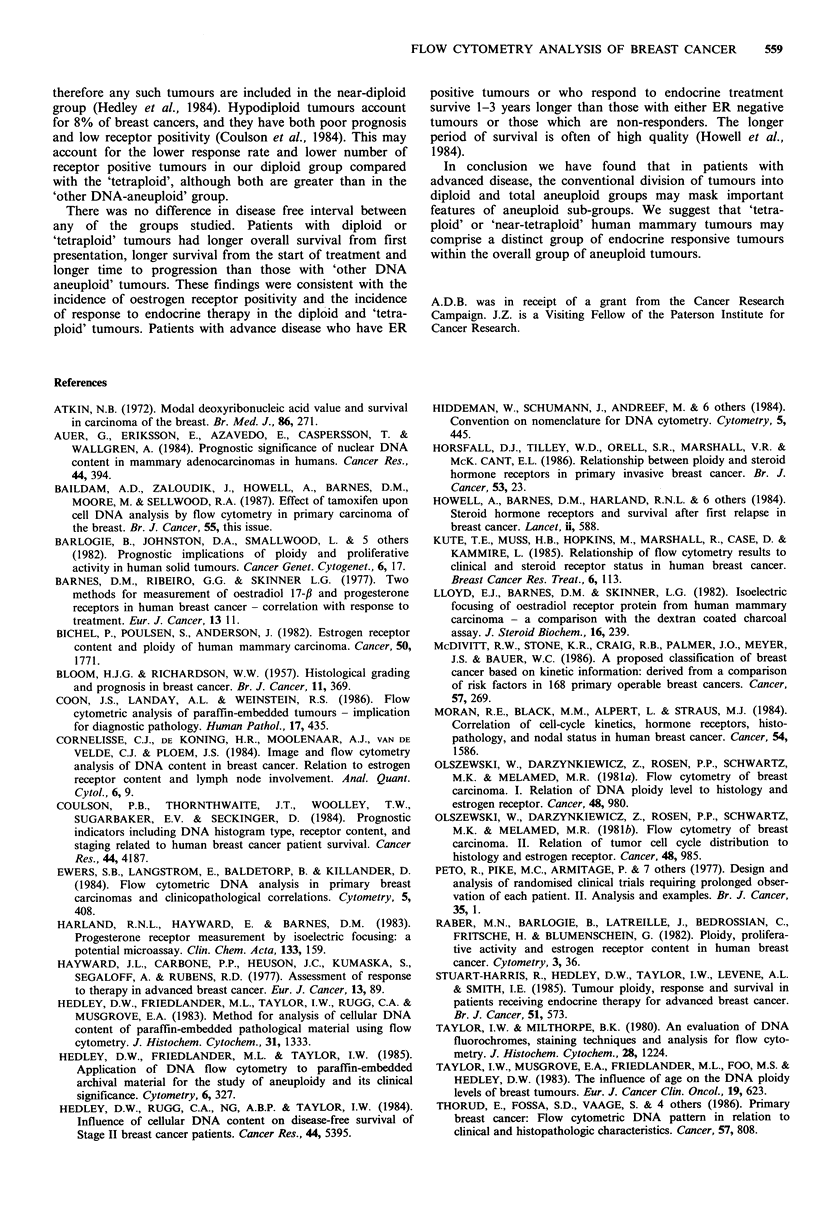# DNA analysis by flow cytometry, response to endocrine treatment and prognosis in advanced carcinoma of the breast.

**DOI:** 10.1038/bjc.1987.113

**Published:** 1987-05

**Authors:** A. D. Baildam, J. Zaloudik, A. Howell, D. M. Barnes, L. Turnbull, R. Swindell, M. Moore, R. A. Sellwood

## Abstract

The relationship between DNA content of mammary cancer and subsequent response to endocrine therapy was studied in 136 patients with advanced disease. All were treated with tamoxifen or ovarian ablation as first-line systemic therapy after relapse and were evaluable for response according to UICC criteria. DNA characterisation by flow cytometry was used on formalin fixed paraffin-embedded samples of tumour. Tumours were grouped according to DNA index into diploid (n = 52, 38%), 'tetraploid' (n = 46, 34%) and 'other DNA-aneuploid' (n = 38, 28%). The highest proportion of oestrogen receptor positive tumours (ER + ve) was found in the 'tetraploid' tumours (38/46, 85%, Chi-square = 8.53, P less than 0.02), and response rates, (SD + PR + CR), were 26/52 (50%), 34/46 (74%), and 15/38 (39%) respectively (Chi-square = 10.88, P less than 0.005). Patients with diploid or 'tetraploid' tumours survived longer and stayed in remission longer than those with 'other DNA-aneuploid' tumours. We suggest that 'tetraploid' or 'near tetraploid' human mammary tumours may comprise a distinct group of endocrine responsive tumours within the overall group of aneuploid tumours. The conventional interpretation of DNA histograms, grouping into diploid and aneuploid, may be masking important features of some tumour groups.


*Br. J. Cancer* (1987), **55**, 553–559 © The Macmillan Press Ltd., 1987

### DNA analysis by flow cytometry, response to endocrine treatment and prognosis in advanced carcinoma of the breast

A.D. Baildam[1,3], J. Zaloudik[6]*, A. Howell[2], D.M. Barnes[3]**, L. Turnbull[4], R. Swindell[5], M. Moore[6] & R.A. Sellwood[1]

*Departments of [1]Surgery, [2]Medical Oncology, [3]Clinical Research, [4]Pathology, [5]Medical Statistics and [6]Paterson Institute for Cancer Research, Christie Hospital and Holt Radium Institute, Manchester M20 9BX, UK.*

**Summary** The relationship between DNA content of mammary cancer and subsequent response to endocrine therapy was studied in 136 patients with advanced disease. All were treated with tamoxifen or ovarian ablation as first-line systemic therapy after relapse and were evaluable for response according to UICC criteria. DNA characterisation by flow cytometry was used on formalin fixed paraffin-embedded samples of tumour. Tumours were grouped according to DNA index into diploid ($n=52$, 38%), 'tetraploid' ($n=46$, 34%) and 'other DNA-aneuploid' ($n=38$, 28%). The highest proportion of oestrogen receptor positive tumours (ER+ve) was found in the 'tetraploid' tumours (38/46, 85%, Chi-square = 8.53, $P<0.02$), and response rates, (SD+PR+CR), were 26/52 (50%), 34/46 (74%), and 15/38 (39%) respectively (Chi-square = 10.88, $P<0.005$). Patients with diploid or 'tetraploid' tumours survived longer and stayed in remission longer than those with 'other DNA-aneuploid' tumours. We suggest that 'tetraploid' or 'near tetraploid' human mammary tumours may comprise a distinct group of endocrine responsive tumours within the overall group of aneuploid tumours. The conventional interpretation of DNA histograms, grouping into diploid and aneuploid, may be masking important features of some tumour groups.

In the past cellular DNA ploidy was assessed by the use of nucleic acid specific stains on tissue sections and microdensitometry. Only relatively small numbers of cells could be assessed. The development of flow cytometry (FCM) has brought a rapid and reproducible technique for determination of DNA content of tumours. Thousands of nuclei can be evaluated, and DNA histograms produced which indicate different cell populations. The introduction of a method whereby paraffin-embedded tumours may be used for flow cytometric analysis has led to the examination of archival material, and a means by which cellular DNA content can be related to subsequent response to treatment, survival and a variety of other factors (Hedley *et al.*, 1983, 1985; Coon *et al.*, 1986).

The manner in which studies have been reported conventionally divided tumours into 'diploid' when the DNA content of the cells is normal or near-normal, and 'aneuploid' when the content is clearly abnormal. Two thirds (range 54–92%) of human mammary tumours have been reported to be aneuploid (Olszewski *et al.*, 1981*a*; Barlogie *et al.*, 1982; Raber *et al.*, 1982; Cornelisse *et al.*, 1984; Ewers *et al.*, 1984; Moran *et al.*, 1984; Hedley *et al.*, 1984, 1985; McDivitt *et al.*, 1986; Thorud *et al.*, 1986; Horsfall *et al.*, 1986).

The results published so far are confusing. Findings obtained with static cytometry suggest that patients with diploid tumours survive longer than those with aneuploid ones (Atkin *et al.*, 1972; Auer *et al.*, 1984), and a similar trend has been found in one study with flow cytometry (Coulson *et al.*, 1984). One report indicates that aneuploid tumours recur more rapidly than diploid tumours (Ewers *et al.*, 1984), but survival of patients after relapse is apparently not related to DNA ploidy (Hedley *et al.*, 1984; Stuart-Harris *et al.*, 1985). Aneuploidy has been associated with lymph node metastases (Barlogie *et al.*, 1982; Cornelisse *et al.*, 1984), but others have failed to confirm the relationship (Taylor *et al.*, 1983; Moran *et al.*, 1984).

There is a consistent trend for a greater proportion of diploid tumours to be oestrogen receptor positive when compared to aneuploid tumours; this difference achieves statistical significance in some studies (Bichel *et al.*, 1982; Cornelisse *et al.*, 1984; Coulson *et al.*, 1984; Horsfall *et al.*, 1986) but not in others (Olszewski *et al.*, 1981*a*; Raber *et al.*, 1982; Taylor *et al.*, 1983; Hedley *et al.*, 1984; McDivitt *et al.*, 1985; Kute *et al.*, 1985).

In most series tetraploid tumours are included within the total aneuploid group, but it is possible that not all components of the total aneuploid group may behave in the same way. In one study with static cytometry, longer survival was found for patients with diploid or tetraploid tumours when compared with the remainder, and this suggests that further work is required to determine the significance of some aneuploid subgroups (Auer *et al.*, 1984).

In this study we have related receptor status, response to endocrine therapy and survival from the start of endocrine treatment to DNA content of tumour cells. Because of the contradictory reports which result when tumours are grouped only as either diploid or aneuploid, the analyses were performed by DNA index, so that characteristics of aneuploid sub-groups could be investigated separately (Coulson *et al.*, 1984; Hiddeman *et al.*, 1984).

### Patients and methods

### *Patients*

This study was carried out on 136 patients who were treated with endocrine therapy as first systemic treatment for advanced mammary cancer, between 1975 and 1983, at the University Hospital of South Manchester. They were chosen because none had had any previous systemic treatment, all were evaluable for response to endocrine manipulation according to internationally accepted criteria (Hayward *et al.*, 1977), and all had had tumour steroid hormone receptors measured.

Response was assessed as complete (CR) when all measurable disease disappeared, partial (PR) when the size of measurable lesions decreased >50% in at least two planes, and static (SD) when the disease remained unchanged for a minimum of 6 months. Non-responding tumours were categorised as progressive disease (PD). SD

*Present address: Surgical Department, Institute of Clinical and Experimental Oncology, Zluty Kopec 7, 60200 Brno, Czechoslovakia.
**Present address: Department of Oncology, Hedley Atkins Unit Laboratory, 2nd floor, New Guy's House, Guy's Hospital, London SE1 9RT.
Correspondence: A.D. Baildam.
Received 20 August 1986; and in revised form, 19 December 1986.

was included with PR and CR as a positive response, because patients with SD fared just as well as those with PR.

At the start of endocrine therapy 117 patients were post-menopausal and were treated with tamoxifen 20 mg $day^{-1}$; 19 were premenopausal and treated by ovarian ablation. Forty patients (29%), presented with locally advanced disease, and 96 (71%) with recurrent disease after previous surgery: they had a median disease free interval of 21 months. Clinical characteristics of these 136 are shown (Table I). Full blood count, serum biochemistry and radiography of the chest, lumbar spine and pelvis were performed regularly. Isotope bone scans were performed on entry, and again at 6–12 month intervals. Disease free interval (DFI), time to progression and survival were recorded.

*Flow cytometry*

Paraffin-embedded, formalin-fixed material was used throughout. Blocks from the primary tumour ($n=125$) or from a skin metastasis ($n=11$) were sectioned. Nuclear suspensions for FCM analysis were prepared by the method of Hedley *et al.*, 1983, but with a slight modification of the fluorochrome. A single 30 $\mu$m section was dewaxed twice for 10 min in xylene, the rehydrated in reducing concentrations of ethanol (100%, 95%, 70%, 50%) and washed in tris-buffered saline (TBS), for 10 min. The rehydrated sections were incubated for 1 h in RNase (Sigma Co.) at a concentration of 1 mg $ml^{-1}$ and then exposed for 30 min to 0.5% pepsin adjusted by HCl to pH 1.5. Release of nuclei was improved by vortex mixing or by gentle repeated aspiration. Suspensions were centrifuged for 15 min and pellets resuspended after filtration through nylon mesh in propidium iodide staining solution (0.05 mg $ml^{-1}$ in 1.12% sodium citrate). Measurements were obtained from a cytofluorograph model 4800A (Biophysics System Inc, Mahopac, New Jersey, USA) with argon laster interfaced to a Hewlett-Packard 9845A Desk Top Computer. Five thousand nuclei were measured from each sample.

*Analysis of histograms*

Half peak coefficient of variability (CV) was evaluated for each histogram and ranged from 2–10% (median 5%). For the description of aneuploid peaks, DNA indices were used, calculated as the ratio of the aneuploid peak channel to the first peak, which was considered to be diploid or near diploid and was present on each histogram (Coulson *et al.*, 1984). Diploid tumours were defined by DNA index 1.0–1.1, 'tetraploid' tumours by a diploid G2+M/tetraploid G1 ≧ 10% nuclei analysed, together with the presence of a tetraploid G2+M peak. In multiploid tumours each peak was defined by its own DNA index and the greatest one used for the overall analysis. For calibration of the cytofluorograph normal human peripheral blood lymphocytes, fixed in 95% ethanol, and paraffin-embedded tonsils were used. These gave comparable fluorescence for diploid histograms.

*Steroid hormone receptor assays*

Tissue was stored in liquid nitrogen until the time of receptor assay. The frozen sample was homogenised by means of a Teflon capsule and tungsten ball which had been pre-cooled with liquid nitrogen, and subjected to the action of a dismembranator for 30 sec. The resulting powder was suspended in buffer (10mM Tris-HCl pH 7.4 with 1mM EDTA, 0.5mM dithiothreitol and 30% v/v glycerol), and centrifuged at 1,000 *g* for 10 min at 4°C to remove nuclei, fat, and any debris.

For samples of weight greater than 200 mg the dextran coated charcoal assay was used to measure ER and PR: values ≧ 5 fmol $mg^{-1}$ cytosol protein were taken as positive (Barnes *et al.*, 1977). For smaller samples the method of isoelectric focusing (IEF) was employed (Lloyd *et al.*, 1982; Harland *et al.*, 1983). Any positive value with an appropriate isoelectric point (pI) was taken to indicate a positive receptor value. In both assays the total protein content was measured by the BCA protein assay reagent (Pierce UK Ltd., Cambridge, UK).

*Histopathology*

Sections of tumour were processed routinely by a single pathology laboratory. Tissue was fixed for up to 48 h in buffered formalin, and subsequently embedded in paraplast medium on a Shandon tissue processor. Representative blocks were sectioned at a thickness of 4 $\mu$m, mounted on glass slides, stained with haematoxylin and eosin, cleared and mounted with glass coverslips. Each section was adjacent to the section that was taken for FCM analysis, and was reviewed in a coded and randomised order by an experienced single pathologist (LT). Sections in which the area of tumour was <20% were discarded. The type of tumour, the presence or absence of elastosis and invasion of blood vessels and lymphatics was noted. Infiltrating duct carcinomas were graded (Bloom & Richardson, 1957).

*Statistical methods*

All evaluations were made by the Chi-squared and Fisher's exact tests when relating DNA ploidy to other variables. Survival, time to progression, disease free interval and survival from the start of treatment were calculated by the life-table method. The log-rank test and Cox regression analysis were used for comparisons between groups (Peto *et al.*, 1977).

### Results

The major histopathological type, infiltrating duct carcinoma, accounted for 123 of 136 (90%) tumours. There were 8 (6%) cases of infiltrating lobular carcinoma, and 5 (4%) of mucoid carcinoma. Lymphatic or vascular invasion was apparent in 37 (27%) and the presence of elastosis, in 23 (17%). The frequency of DNA indices is shown (Figure 1).

Categorised into diploid (52 of 136, 38%) and total aneuploid (84 of 136, 62%), there was no statistically significant difference between these two groups with regard to any of the other tumour or patient variables, including histopathology, receptor status, response to endocrine therapy, and survival. There was also no difference with regard to DFI, survival from presentation, survival from the start of treatment or time to progression (Figures 2, 3, 4 and 5).

Evaluations were repeated with the DNA histograms grouped according to DNA index, to examine more closely aneuploid sub-groups. Of the 136 tumours studied, 52 (40%) had a DNA index of ≦1.1 and were regarded as diploid or near-diploid, 46 (34%) had DNA indices between 1.8 and 2.0: 19 (14%) were near-tetraploid with DNA index 1.8–1.9, and 27 (20%), were tetraploid with an index of 2.0. The remainder, 'other DNA-aneuploid', had DNA indices between 1.2 and 1.7 or ≧2.1.

The highest proportion of oestrogen receptor positive tumours was found in tumours with near-tetraploid DNA indices between 1.8 and 1.9 (17 of 19, 90% ER positive) and the tetraploid index of 2.0 (21 of 27, 81% ER positive) (Table II).

The highest proportion of responders to endocrine therapy was also found in these two subgroups (16 of 19, 84%, and 18 of 27, 67%, respectively. Table III). The range 1.8–2.0 was combined as the 'tetraploid' group, and compared with the diploid and 'other DNA-aneuploid' groups. The difference between the three groups for ER positivity was highly significant (Chi-square = 8.53, $P<0.02$). Of the 'tetraploid' tumours, 38 of 46 (83%) were ER positive, compared with 35 of 52 (69%) of the diploid tumours, and

**Table I** Clinical characteristics

| | | |
|---|---|---|
| TOTAL | $n=136$ | |
| Median age | 62 years | (Range 25–87 years) |
| Menopausal status | | |
| Premenopausal | 19 | (14%) |
| Postmenopausal | 117 | (86%) |
| Inoperable primary tumour | 40 | (29%) |
| Metastatic disease | 96 | (71%) |
| Original tumour size | | |
| T0 | 1 | (<1%) |
| T1 | 11 | (8%) |
| T2 | 60 | (44%) |
| T3 | 22 | (16%) |
| T4 | 42 | (31%) |
| Sites of disease (>1 site in some patients) | | |
| Soft tissue | 101 | (73%) |
| Bone | 57 | (42%) |
| Lung | 41 | (30%) |
| Liver | 5 | (4%) |
| Treatment | | |
| Tamoxifen | 117 | (86%) |
| Ovarian ablation | 19 | (14%) |

**Figure 1** Frequency of DNA indices.

**Figure 2** Survival from presentation – diploid *vs.* total aneuploid.

22 of 38 (57%) of the 'other DNA-aneuploid' tumours (Table IV).

There was also a significant difference for response to endocrine treatment: (Chi-square = 10.88, $P<0.005$). Of the 'tetraploid' tumours 34 of 46 (74%) responded, and 20 of these 34 (59%) were complete or partial responses. Diploid

**Figure 3** Disease free interval – diploid *vs.* total aneuploid.

**Figure 4** Time to progression from the start of treatment – diploid *vs.* total aneuploid.

**Figure 5** Survival from the start of treatment – diploid *vs.* total aneuploid.

**Table II** DNA index and receptor status

| DNA index | n= | ER+ve | PR+ve |
|---|---|---|---|
| ≦1.1 | 52 | 35 (69%) | 28 (55%) |
| 1.2–1.7 | 27 | 16 (59%) | 14 (52%) |
| 1.8–1.9 | 19 | 17 (90%) | 11 (58%) |
| 2.0 | 27 | 21 (81%) | 16 (62%) |
| ≧2.1 | 11 | 6 (54%) | 4 (36%) |
| | | **$P<0.02$** | $P=0.51$ |

**Table III** DNA index and response to endocrine therapy

| DNA index | n= | CR+PR | CR+PR+SD | PD |
|---|---|---|---|---|
| ≦1.1 | 52 | 16 (30%) | 26 (50%) | 26 (50%) |
| 1.2–1.7 | 27 | 7 (26%) | 12 (44%) | 15 (54%) |
| 1.8–1.9 | 19 | 9 (47%) | 16 (85%) | 3 (15%) |
| 2.0 | 27 | 11 (41%) | 18 (67%) | 9 (23%) |
| ≧2.1 | 11 | 2 (18%) | 3 (27%) | 8 (67%) |
| | | **$P<0.04$** | **$P<0.01$** | **$P<0.01$** |

**Table IV** DNA ploidy and receptor status

| | n= | ER+ve | PR+ve |
|---|---|---|---|
| Diploid | 52 | 35 (69%) | 28 (55%) |
| 'Tetraploid'[a] | 46 | 38 (83%) | 27 (60%) |
| 'Other DNA-aneuploid'[b] | 38 | 22 (57%) | 18 (47%) |
| | | Chi-square =8.53 | Chi-square =1.62 |
| | | **$P<0.02$** | **$P<0.40$** |

[a]DNA indices 1.8–2.0 ('tetraploid'); [b]1.2–1.7 with ≧2.1 ('other DNA-aneuploid').

**Table V** DNA ploidy and response to endocrine therapy

| | n= | CR+PR | CR+PR+SD | PD |
|---|---|---|---|---|
| Diploid | 52 | 16 (30%) | 26 (50%) | 26 (50%) |
| 'Tetraploid'[a] | 46 | 20 (43%) | 34 (74%) | 12 (26%) |
| 'Other DNA-aneuploid'[b] | 38 | 9 (24%) | 15 (39%) | 23 (61%) |
| | | **$P<0.05$** | **$P<0.007$** | **$P<0.007$** |

[a]DNA indices 1.8–2.0 ('tetraploid'); [b]1.2–1.7 with ≧2.1 ('other DNA-aneuploid')

tumours had a 50% response rate, and 'other DNA-aneuploid' tumours had a 39% response rate (Table V).

DNA ploidy had no influence on histopathological type (Table VI). There was an insignificant trend for 'other DNA-aneuploid' tumours to be of higher grade than diploid or 'tetraploid' tumours: 10 of 33 (30%) of 'other DNA-aneuploid' tumours were grade III, compared with 13 of 81 (16%) of diploid or 'tetraploid' tumours. There was also an

**Table VI** DNA ploidy and histopathology type

| | IDC | Grade[a] I | II | III | ILC | Mucoid |
|---|---|---|---|---|---|---|
| Diploid | 49 | 8 | 27 | 5 | 3 | 0 |
| 'Tetraploid' | 40 | 9 | 24 | 7 | 3 | 3 |
| 'Other DNA-aneuploid' | 34 | 4 | 19 | 10 | 2 | 2 |

IDC = infiltrating duct carcinoma, ILC = infiltrating lobular carcinoma.
[a]Grade assessed on 113 of 123 infiltrating duct carcinomas. $P$ = NS for all variables.

insignificant trend for 'other DNA-aneuploid' tumours to have a greater degree of nuclear pleomorphism and higher number of mitoses than diploid and 'tetraploid' tumours (Table VII). There was no difference in the incidence of lymphatic or vascular invasion between the three groups, but there was a trend, although still insignificant, for a higher incidence of elastosis in 'tetraploid' tumours (10 of 41, 25%), than in 'other DNA-aneuploid' tumours (5 of 33, 15%) (Table VII).

Patients with diploid and 'tetraploid' tumours had a trend for increased overall survival ($P>0.06$, Figure 6), a longer time to progression on endocrine therapy ($P<0.001$, Figure 7) and a trend for increased survival from the start of treatment when compared with patients with 'other DNA-aneuploid' tumours (Figure 8). There was no significant difference in the disease free interval between the three groups – the increased survival and longer time to progression for patients with diploid or 'tetraploid' tumours related to the period after recurrence and institution of therapy (Figure 9). When the diploid and 'tetraploid' groups were combined there was a greater overall survival ($P<0.03$, Figure 10), time to progression ($P<0.01$, Figure 11) and trend for survival from the start of treatment (Figure 12). But there remained no difference in the disease-free interval between the groups (Figure 13).

**Figure 6** Survival from presentation – diploid vs. 'tetraploid' vs. 'other DNA-aneuploid'.

**Table VII** DNA ploidy and histopathological variables

| | Nuclear grade I | II | III | Mitosis score I | II | III | Elastosis present | Lymph./Vasc. invasion present |
|---|---|---|---|---|---|---|---|---|
| Diploid | 7 | 29 | 4 | 25 | 13 | 2 | 8 (18%) | 14 (32%) |
| 'Tetraploid' | 4 | 28 | 9 | 23 | 14 | 4 | 10 (25%) | 14 (34%) |
| 'Other DNA-aneuploid' | 3 | 21 | 9 | 13 | 15 | 5 | 5 (15%) | 9 (30%) |

$P$ = NS for all comparisons.

**Figure 7** Time to progression from the start of treatment – diploid *vs.* 'tetraploid' *vs.* 'other DNA-aneuploid'.

**Figure 8** Survival from the start of treatment – diploid *vs.* 'tetraploid' *vs.* 'other DNA-aneuploid'.

**Figure 9** Disease free interval – diploid *vs.* 'tetraploid' *vs.* 'other DNA-aneuploid'.

**Figure 10** Survival from presentation – diploid plus 'tetraploid' *vs.* 'other DNA-aneuploid'.

**Figure 11** Time to progression from the start of treatment – diploid plus 'tetraploid' *vs.* 'other DNA-aneuploid'.

**Figure 12** Survival from the start of treatment – diploid plus 'tetraploid' *vs.* 'other DNA-aneuploid'.

**Figure 13** Disease free interval – diploid plus 'tetraploid' *vs* 'other DNA-aneuploid'.

### Discussion

Flow cytometric DNA analysis is rapid, objective, reproducible and feasible for the routine examination not only of fresh tissue but also of fixed paraffin embedded samples (Hedley *et al.*, 1983, 1985; Coon *et al.*, 1986). The use of standards is limited on paraffin-embedded material because of the quality of fixation and possibly also on the age of the blocks (Hedley *et al.*, 1983). In this study normal peripheral human lymphocytes and tonsil tissue blocks were used to generate normal diploid DNA histogram patterns for calibration and comparison for other histograms. The advantages and drawbacks of paraffin-embedded tissue for flow cytometry have been discussed fully elsewhere (Hedley *et al.*, 1985). Fresh tissue is usually not available when a retrospective clinical study is being performed.

The cytofluorograph 4800A interfaced to the computer with an established program allowed either 5,000 or 10,000 nuclei to be analysed. We did not find any difference for interpretation between these two options, and from a statistical point of view 5,000 nuclei were sufficient to plot the distribution.

The results obtained with propidium iodide, a non-specific nucleic acid stain, are comparable after ribonuclease pre-treatment with the DNA-specific stain 4′,6′-diamidino-2-phenylindole (DAPI) (Taylor *et al.*, 1980). In the initial stages of this work we treated samples either before or after pepsin digestion with RNase. Because the results were comparable, we preferred the technically simpler procedure of RNase treatment before pepsin.

Many studies have attempted to relate DNA ploidy to clinical and histological features, but the results have been disappointing (Olszewski *et al.*, 1981*a*; Cornelisse *et al.*, 1984; Horsfall *et al.*, 1984; Coulson *et al.*, 1984; Kute *et al.*, 1985; Stuart-Harris *et al.*, 1985). Characterised conventionally into diploid and aneuploid, DNA ploidy analysis at present neither adds to knowledge of tumour prognosis nor influences therapy for an individual patient. Conventionally the majority of breast carcinomas are described as aneuploid. We found no difference between the diploid and *total* aneuploid groups of tumours with respect to any other variable, including histopathology, hormone receptor status, response to endocrine therapy, and survival.

When the total aneuploid group was sub-divided into 'tetraploid' and 'other DNA-aneuploid' tumours, still no clear-cut differences emerged with regard to histopathology. There was a trend for diploid and 'tetraploid' tumours to be of lower grade, lower mitosis score, and to have elastosis present more frequently when compared with 'other DNA-aneuploid' tumours, but this trend failed to achieve statistical significance. These findings are in accord with those of others: some studies found no association between histological differentiation and ploidy (Kute *et al.*, 1985; Taylor *et al.*, 1983), whereas others found an insignificant trend for diploid tumours to be of lower grade than the total group of aneuploid tumours (Thorud *et al.*, 1984; Moran *et al.*, 1984; Olszewski *et al.*, 1981*a*, *b*).

Stuart-Harris *et al.* (1985) related response to treatment with DNA ploidy in 42 endocrine treated evaluable patients, and found no significant difference in response between the diploid and aneuploid groups; there was no attempt to subdivide the aneuploid group. In our study it was only when the total aneuploid group was divided into 'tetraploid' and 'other DNA-aneuploid' tumours that differences emerged. The results indicate that 'tetraploid' or 'near-tetraploid' tumours (DNA index 1.8–2.0) had a greater probability of containing oestrogen receptors (85% were ER positive), and of being responsive to endocrine therapy (74% responded) than 'other DNA-aneuploid' tumours (57% were ER positive. 39% responded). In addition patients with 'tetraploid' tumours survived longer and remained in remission on endocrine therapy longer than those with 'other DNA-aneuploid' tumours.

The definition of tetraploid peaks is an area of difficult interpretation. Most authors do not define tetraploidy, because they choose to distinguish only diploid from non-diploid tumours. In one study by static cytometry, 50% (18 of 36) of good prognosis tumours were tetraploid, whereas only 9% (10 of 42) of a poor prognosis group were tetraploid (Auer *et al.*, 1984). In the largest series published on flow cytometry in breast cancer, the indidence of tetraploid tumours reported is significantly lower than ours (Ewers *et al.*, 1984). The probable explanation lies in interpretation. In that study, tetraploid was defined as a diploid G2+M/tetraploid G1 peak representing $\geqq$20% of analysed nuclei, together with the presence of a tetraploid G2+M peak. In our study we included as 'tetraploid' histograms with diploid G2+M/tetraploid G1 peaks $\geqq$10% of analysed nuclei, together with the presence of a tetraploid G2+M peak. We widened the criteria because it is impossible to distinguish diploid from tetraploid tumours by DNA histogram analysis alone, and doubt has been expressed with regard to the validity of using the figure of 20% as the threshold (Ewers *et al.*, 1984). We used a cut-off point of 10%, because that was the threshold at which tetraploid G2+M peaks were apparent, at a position corresponding to the approximate position of DNA index of 4.0. We do not suppose that clumping of nuclei was significant in our study, because there was no significant occurrence of sextaploid indices (triplets), which should be present also if doublets have been produced by clumping. Internationally accepted nomenclature for flow cytometry does not include a definition of tetraploid (Hiddemann *et al.*, 1984). We included tumours with histograms of DNA index 1.8–2.0, as 'tetraploid' in order to accommodate the coefficients of variation ($\leqq$10%) in DNA index which can occur with the use of paraffin-embedded tissue (Hedley *et al.*, 1983, 1985). Furthermore we evaluated them together because hypothetically they may be generated by similar mechanisms (Baildam *et al.*, 1987).

Auer *et al.* (1984) used the technique of static cytometry in breast cancer, and in that series the majority of patients who survived 15 years or more from diagnosis had diploid or tetraploid tumours. Most who died within 2 years had tumours that were hyperdiploid, hypertetraploid, or hypodiploid. This was confirmed by Coulson *et al.* (1984) with flow cytometry on fresh tissue: 22 of 24 (92%) patients who died during the 36 month follow up period had tumours classified into one of these three 'other DNA-aneuploid' groups.

The method of analysis on paraffin-embedded material does not allow the accurate characterisation of hypodiploidy; therefore any such tumours are included in the near-diploid group (Hedley *et al.*, 1984). Hypodiploid tumours account for 8% of breast cancers, and they have both poor prognosis and low receptor positivity (Coulson *et al.*, 1984). This may account for the lower response rate and lower number of receptor positive tumours in our diploid group compared with the 'tetraploid', although both are greater than in the 'other DNA-aneuploid' group.

There was no difference in disease free interval between any of the groups studied. Patients with diploid or 'tetraploid' tumours had longer overall survival from first presentation, longer survival from the start of treatment and longer time to progression than those with 'other DNA aneuploid' tumours. These findings were consistent with the incidence of oestrogen receptor positivity and the incidence of response to endocrine therapy in the diploid and 'tetraploid' tumours. Patients with advance disease who have ER positive tumours or who respond to endocrine treatment survive 1–3 years longer than those with either ER negative tumours or those which are non-responders. The longer period of survival is often of high quality (Howell *et al.*, 1984).

In conclusion we have found that in patients with advanced disease, the conventional division of tumours into diploid and total aneuploid groups may mask important features of aneuploid sub-groups. We suggest that 'tetraploid' or 'near-tetraploid' human mammary tumours may comprise a distinct group of endocrine responsive tumours within the overall group of aneuploid tumours.

A.D.B. was in receipt of a grant from the Cancer Research Campaign. J.Z. is a Visiting Fellow of the Paterson Institute for Cancer Research.

### References

ATKIN, N.B. (1972). Modal deoxyribonucleic acid value and survival in carcinoma of the breast. *Br. Med. J.*, **86**, 271.

AUER, G., ERIKSSON, E., AZAVEDO, E., CASPERSSON, T. & WALLGREN, A. (1984). Prognostic significance of nuclear DNA content in mammary adenocarcinomas in humans. *Cancer Res.*, **44**, 394.

BAILDAM, A.D., ZALOUDIK, J., HOWELL, A., BARNES, D.M., MOORE, M. & SELLWOOD, R.A. (1987). Effect of tamoxifen upon cell DNA analysis by flow cytometry in primary carcinoma of the breast. *Br. J. Cancer*, **55**, this issue.

BARLOGIE, B., JOHNSTON, D.A., SMALLWOOD, L. & 5 others (1982). Prognostic implications of ploidy and proliferative activity in human solid tumours. *Cancer Genet. Cytogenet.*, **6**, 17.

BARNES, D.M., RIBEIRO, G.G. & SKINNER L.G. (1977). Two methods for measurement of oestradiol 17-$\beta$ and progesterone receptors in human breast cancer – correlation with response to treatment. *Eur. J. Cancer*, **13** 11.

BICHEL, P., POULSEN, S., ANDERSON, J. (1982). Estrogen receptor content and ploidy of human mammary carcinoma. *Cancer*, **50**, 1771.

BLOOM, H.J.G. & RICHARDSON, W.W. (1957). Histological grading and prognosis in breast cancer. *Br. J. Cancer*, **11**, 369.

COON, J.S., LANDAY, A.L. & WEINSTEIN, R.S. (1986). Flow cytometric analysis of paraffin-embedded tumours – implication for diagnostic pathology. *Human Pathol.*, **17**, 435.

CORNELISSE, C.J., DE KONING, H.R., MOOLENAAR, A.J., VAN DE VELDE, C.J. & PLOEM, J.S. (1984). Image and flow cytometry analysis of DNA content in breast cancer. Relation to estrogen receptor content and lymph node involvement. *Anal. Quant. Cytol.*, **6**, 9.

COULSON, P.B., THORNTHWAITE, J.T., WOOLLEY, T.W., SUGARBAKER, E.V. & SECKINGER, D. (1984). Prognostic indicators including DNA histogram type, receptor content, and staging related to human breast cancer patient survival. *Cancer Res.*, **44**, 4187.

EWERS, S.B., LANGSTROM, E., BALDETORP, B. & KILLANDER, D. (1984). Flow cytometric DNA analysis in primary breast carcinomas and clinicopathological correlations. *Cytometry*, **5**, 408.

HARLAND, R.N.L., HAYWARD, E. & BARNES, D.M. (1983). Progesterone receptor measurement by isoelectric focusing: a potential microassay. *Clin. Chem. Acta*, **133**, 159.

HAYWARD, J.L., CARBONE, P.P., HEUSON, J.C., KUMASKA, S., SEGALOFF, A. & RUBENS, R.D. (1977). Assessment of response to therapy in advanced breast cancer. *Eur. J. Cancer*, **13**, 89.

HEDLEY, D.W., FRIEDLANDER, M.L., TAYLOR, I.W., RUGG, C.A. & MUSGROVE, E.A. (1983). Method for analysis of cellular DNA content of paraffin-embedded pathological material using flow cytometry. *J. Histochem. Cytochem.*, **31**, 1333.

HEDLEY, D.W., FRIEDLANDER, M.L. & TAYLOR, I.W. (1985). Application of DNA flow cytometry to paraffin-embedded archival material for the study of aneuploidy and its clinical significance. *Cytometry*, **6**, 327.

HEDLEY, D.W., RUGG, C.A., NG, A.B.P. & TAYLOR, I.W. (1984). Influence of cellular DNA content on disease-free survival of Stage II breast cancer patients. *Cancer Res.*, **44**, 5395.

HIDDEMAN, W., SCHUMANN, J., ANDREEF, M. & 6 others (1984). Convention on nomenclature for DNA cytometry. *Cytometry*, **5**, 445.

HORSFALL, D.J., TILLEY, W.D., ORELL, S.R., MARSHALL, V.R. & McK. CANT, E.L. (1986). Relationship between ploidy and steroid hormone receptors in primary invasive breast cancer. *Br. J. Cancer*, **53**, 23.

HOWELL, A., BARNES, D.M., HARLAND, R.N.L. & 6 others (1984). Steroid hormone receptors and survival after first relapse in breast cancer. *Lancet*, **ii**, 588.

KUTE, T.E., MUSS, H.B., HOPKINS, M., MARSHALL, R., CASE, D. & KAMMIRE, L. (1985). Relationship of flow cytometry results to clinical and steroid receptor status in human breast cancer. *Breast Cancer Res. Treat.*, **6**, 113.

LLOYD, E.J., BARNES, D.M. & SKINNER, L.G. (1982). Isoelectric focusing of oestradiol receptor protein from human mammary carcinoma – a comparison with the dextran coated charcoal assay. *J. Steroid Biochem.*, **16**, 239.

McDIVITT, R.W., STONE, K.R., CRAIG, R.B., PALMER, J.O., MEYER, J.S. & BAUER, W.C. (1986). A proposed classification of breast cancer based on kinetic information: derived from a comparison of risk factors in 168 primary operable breast cancers. *Cancer*, **57**, 269.

MORAN, R.E., BLACK, M.M., ALPERT, L. & STRAUS, M.J. (1984). Correlation of cell-cycle kinetics, hormone receptors, histopathology, and nodal status in human breast cancer. *Cancer*, **54**, 1586.

OLSZEWSKI, W., DARZYNKIEWICZ, Z., ROSEN, P.P., SCHWARTZ, M.K. & MELAMED, M.R. (1981*a*). Flow cytometry of breast carcinoma. I. Relation of DNA ploidy level to histology and estrogen receptor. *Cancer*, **48**, 980.

OLSZEWSKI, W., DARZYNKIEWICZ, Z., ROSEN, P.P., SCHWARTZ, M.K. & MELAMED, M.R. (1981*b*). Flow cytometry of breast carcinoma. II. Relation of tumor cell cycle distribution to histology and estrogen receptor. *Cancer*, **48**, 985.

PETO, R., PIKE, M.C., ARMITAGE, P. & 7 others (1977). Design and analysis of randomised clinical trials requiring prolonged observation of each patient. II. Analysis and examples. *Br. J. Cancer*, **35**, 1.

RABER, M.N., BARLOGIE, B., LATREILLE, J., BEDROSSIAN, C., FRITSCHE, H. & BLUMENSCHEIN, G. (1982). Ploidy, proliferative activity and estrogen receptor content in human breast cancer. *Cytometry*, **3**, 36.

STUART-HARRIS, R., HEDLEY, D.W., TAYLOR, I.W., LEVENE, A.L. & SMITH, I.E. (1985). Tumour ploidy, response and survival in patients receiving endocrine therapy for advanced breast cancer. *Br. J. Cancer*, **51**, 573.

TAYLOR, I.W. & MILTHORPE, B.K. (1980). An evaluation of DNA fluorochromes, staining techniques and analysis for flow cytometry. *J. Histochem. Cytochem.*, **28**, 1224.

TAYLOR, I.W., MUSGROVE, E.A., FRIEDLANDER, M.L., FOO, M.S. & HEDLEY, D.W. (1983). The influence of age on the DNA ploidy levels of breast tumours. *Eur. J. Cancer Clin. Oncol.*, **19**, 623.

THORUD, E., FOSSA, S.D., VAAGE, S. & 4 others (1986). Primary breast cancer: Flow cytometric DNA pattern in relation to clinical and histopathologic characteristics. *Cancer*, **57**, 808.